# Multi-Institutional Care in Clinical Stage II and III Esophageal Cancer

**DOI:** 10.1016/j.athoracsur.2022.06.049

**Published:** 2022-07-21

**Authors:** Kristen E Rhodin, Vignesh Raman, Christopher W Jensen, Lillian Kang, Daniel P Nussbaum, Betty C Tong, Dan G Blazer, Thomas A D’Amico

**Affiliations:** 1 Department of Surgery, Duke University Medical Center, Durham, NC; 2 Division of Cardiovascular and Thoracic Surgery, Department of Surgery, Duke University Medical Center, Durham, NC

**Keywords:** Esophageal Cancer, Fragmented Care, Multi-Institutional Care, Neoadjuvant Therapy, Esophagectomy

## Abstract

**Objective:**

Management of clinical stage II/III esophageal cancer requires multidisciplinary care. Multi-institutional care has been associated with worse survival in other malignancies. Herein, we aimed to determine the impact of multi-institutional care on survival in patients with stage II/III esophageal cancer.

**Methods:**

The 2004-2016 National Cancer Database (NCDB) was queried for patients with clinical stage II/III esophageal cancer who received neoadjuvant chemotherapy +/− radiation followed by surgical resection. Patients were stratified into two groups: multi-institutional or single institution care. Survival between groups was compared using Kaplan-Meier and multivariable Cox Proportional Hazards methods. Multivariable logistic regression was performed to identify factors associated with multi-institutional care.

**Results:**

Overall, 11,399 patients met study criteria: 6,569 (57.6%) received multi-institutional care and 4,830 (42.4%) received care at a single institution. In a multivariable analysis, factors associated with multi-institutional care were later year of diagnosis, greater distance from treating facility, residing in an urban or rural setting (vs metro), and residing in states without Medicaid expansion. Care at a single institution was associated with Black race, lack of insurance, and treatment at higher volume or academic centers. Despite these differences, patients who received multi-institutional care had comparable survival to those who received care at a single institution (HR 0.97, 95% CI 0.92-1.03; p=0.30).

**Conclusions:**

In this NCDB analysis, multi-institutional care was not associated with inferior overall survival. As complex cancer care becomes more regionalized, patients may consider receiving part of their cancer care closer to home while traveling to surgical centers of excellence should be encouraged.

## Introduction

Regionalization of complex cancer care is becoming increasingly common within the United States.(1, 2) This trend is particularly evident in the setting of complex cancer operations, such as esophagectomy, and many studies have highlighted the volume-outcomes relationship with both improved perioperative outcomes and long-term survival at high-volume centers.(3-8)

In addition to surgery, many gastrointestinal malignancies, including locally advanced esophageal cancer, require multidisciplinary treatment. Indeed, current National Comprehensive Cancer Network (NCCN) guidelines recommend that most patients with clinical stage II and III esophageal cancer receive neoadjuvant chemotherapy with or without radiation prior to definitive surgical resection.(9) In contrast to surgery which occurs at one timepoint with an anticipated hospital stay and recovery, chemotherapy and radiation treatments can span periods of weeks to months. Frequent travel to high-volume centers for these lengthy periods may not be financially nor logistically feasible for all patients.(10) Alternatively, seeking care far from centers of excellence may not lend the multidisciplinary expertise required to receive guideline-concordant care nor a high-quality cancer operation.(4) Consequently, patients with esophageal cancer are vulnerable to receiving components of their care at several different healthcare facilities.

This pattern, previously termed “fragmented” care, or the receipt of cancer care across multiple healthcare centers (multi-institutional), has been described in other malignancies. The existing literature suggests that patients who receive multi-institutional care endure greater healthcare costs, higher rates of unnecessary procedures and treatments, and receive inferior quality of care.(11-13) Despite these negative associations, multi-institutional care does not appear to have a universal impact on overall survival.(14-19) In the context of increasing regionalization of cancer care, multi-institutional care may represent a new form of health disparity and the Institute of Medicine has previously identified this pattern of cancer care as an area for improvement.(20, 21) However, there is a paucity of literature on its impact in the multidisciplinary treatment for esophageal cancer. Therefore, we aimed to characterize patterns of multi-institutional care and its impact on overall survival in patients with stage II and III esophageal cancer.

## Methods

### Data Source

The National Cancer Database (NCDB) was utilized for retrospective analysis. The NCDB is a national clinical oncology database maintained through collaboration between the American College of Surgeons and the American Cancer Society. The NCDB contains information on cancer patients including diagnosis, pathology, treatments, and outcomes. Data from over 1,500 Commission on Cancer (CoC)-accredited facilities is collected by trained registrars using a standardized data dictionary and captures approximately 70-80% of cancer diagnoses in the United States.(22, 23, 24) The NCDB is de-identified and this study was deemed exempt by the Duke University Health System Institutional Review Board.

### Patient Selection

**Figure 1.**
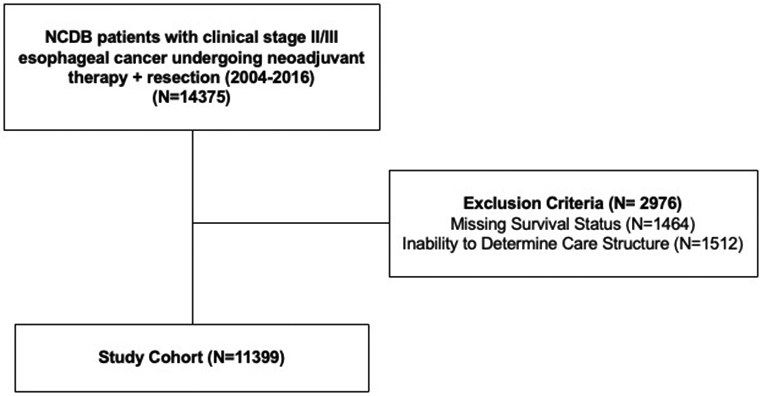
Study Consort Diagram.

The NCDB was queried for patients with clinical stage II and III esophageal cancer (AJCC 8^th^ edition) who received neoadjuvant chemotherapy with or without radiation followed by surgical resection between 2004 and 2016 (Figure 1). Resections with curative intent were included. Patients with missing survival status were excluded.

Care structure was classified as multi-institutional or single institution. Multi-institutional care was defined as receiving a component of their multidisciplinary cancer care (chemotherapy or surgery) at another facility. Patients that received surgery and chemotherapy at the same reporting facility were defined as single institution care. In cases where the reporting facility did not provide either surgery or chemotherapy, care structure could not be determined; therefore, these patients were excluded.

### Statistical Analysis

Patients were stratified by care structure. Baseline demographics for the two groups were calculated and compared using the Pearson’s chi-squared and Wilcoxon rank-sum tests for categorical and continuous variables, respectively. A multivariable logistic regression was performed to identify independent factors associated with the receipt of multi-institutional care. The primary outcome was overall survival, which was analyzed using Kaplan-Meier and multivariable Cox-Proportional Hazards methods. A subgroup analysis was performed on patients receiving multi-institutional care and survival compared among patients undergoing resection at academic or high-volume esophagectomy centers (defined by center esophagectomy volume quartiles in this NCDB cohort). Separate multivariable logistic regression models were created to assess if receipt of multi-institutional care was associated with pathologic upstaging and guideline-concordant nodal harvest as secondary outcomes. All adjusted models incorporated known covariates that were designated *a priori*. Missing data were handled with complete case analysis in regression. All statistical analyses were performed using R version 4.1.1 for Mac (Vienna, Austria) with a designated significance threshold of 0.05 or less.

## Results

### Baseline Patient, Tumor, and Treatment Characteristics

Altogether, 11,399 patients met study criteria with 6,569 (57.6%) receiving multi-institutional care and 4,830 (42.4%) receiving care at a single institution. Baseline patient demographics, tumor, and hospital-level characteristics are shown in Table 1. Patients receiving multi-institutional care were more likely to be older (median age 63 vs 62 years, p=0.028), white (94.8% vs 82.6%, p<0.001), have more recent year of diagnosis (median 2012 vs 2011, p<0.001), and have insurance (98.3% vs 96.8%, p<0.001) compared to those with care at a single institution. These patients were also more likely to reside in states without Medicaid expansion (39.1% vs 32.7%, p<0.001), non-metropolitan areas (21.7% vs 17.9%, p<0.001), and travel farther distances to the reporting facility (median 20.9 vs 15.8 miles, p<0.001). Patients receiving care at a single institution were more commonly treated at academic centers (64.9% vs 50.3%, p<0.001) or those with higher annualized esophagectomy volume (median 8.2 vs 5.2 cases per year, p<0.001). These patients were also more likely to have clinical stage III disease (54.9% vs 52.4%, p=0.007) and squamous histology (19.2% vs 17.1%, p=0.005).

In the overall cohort, median travel distance for chemotherapy was 13.8 miles (IQR 5.9, 32.5) and median travel distance for surgery was 21.3 miles (IQR 8.3, 55.1). The median time interval between initiation of neoadjuvant therapy and surgical resection was longer in those receiving multi-institutional care (95 vs 92 days, p<0.001), although, there was no difference in rates of pathologic upstaging (8.0% vs 7.6%, p=0.41). Patients receiving care at a single institution had higher rates of guideline-concordant lymph node harvest (15 or more nodes) at the time of surgery (40.6% vs 37.7%, p=0.002); however, they had marginally longer post-operative hospital stays (median 10 vs 9 days, p<0.001) and higher rates of readmission (6.1% vs 4.8%, p=0.002).

### Factors Associated with Multi-Institutional Care

**Figure 2.**
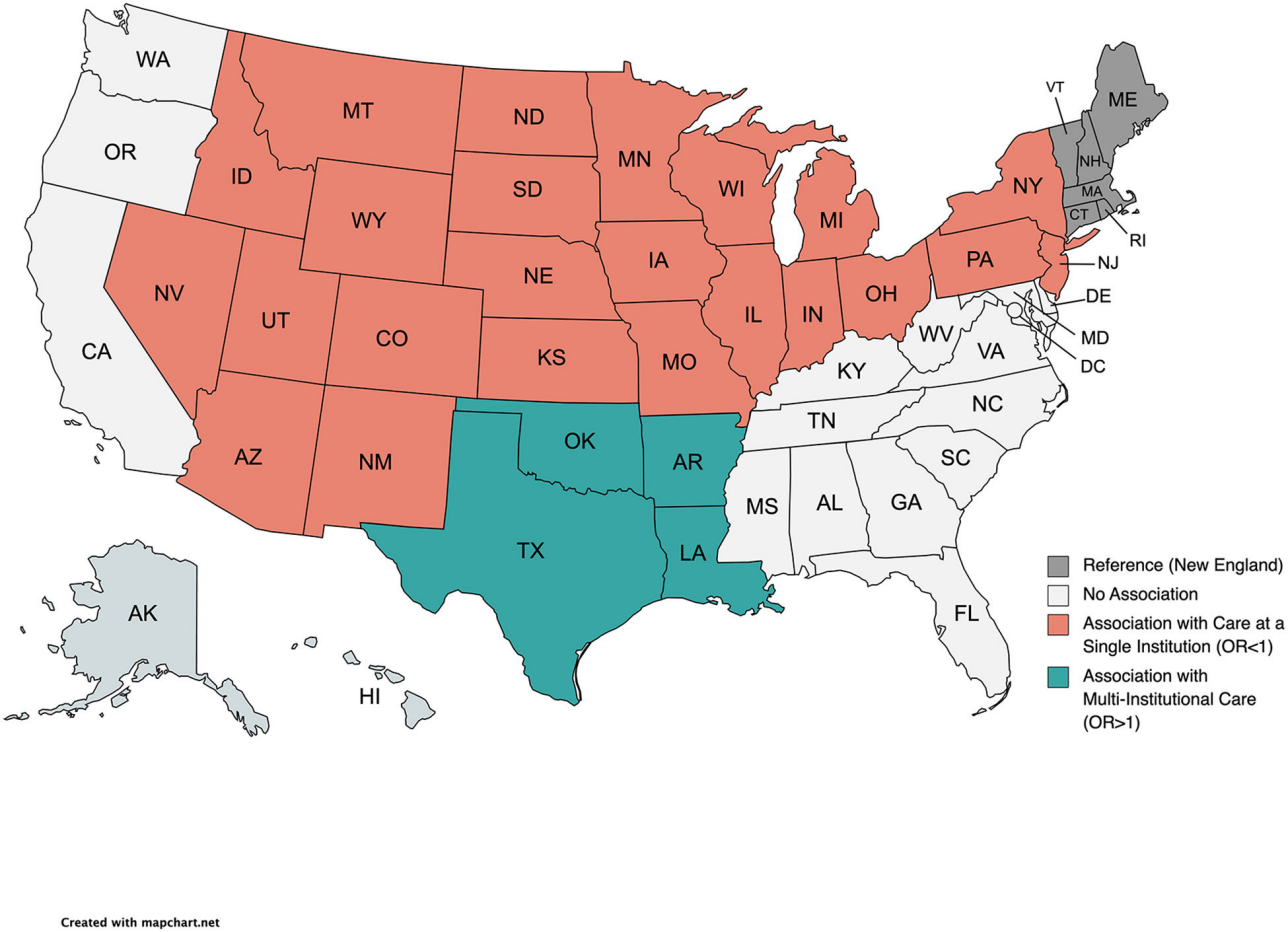
Visual Representation of Association of Geographic Region with Care Structure. Adjusted Odds Ratio (OR) are from the multivariable logistic regression model for factors associated with multi-institutional care. Regions shaded in teal had statistically significant OR greater than 1 indicating association with multi-institutional care; while regions shaded in coral had statistically significant OR less than 1 indicating association with single institution care. The reference region was New England. Created with MapChart.net with a CC BY-SA 4.0 license.

On adjusted analysis, several independent factors were associated with receipt of multi-institutional care. Patient-specific factors included female sex, later year of diagnosis, residing in non-metropolitan areas, geographic regions such as West South Central states (compared to New England) (Figure 2), and increasing travel distance to the reporting facility (Table 2). Conversely, factors associated with receipt of care at a single institution included black race, lack of insurance, squamous histology, residing in a Medicaid expansion state, and states in the Middle Atlantic, East North Central, West North Central, and Mountain regions (Table 2) (Figure 2). Treatment at an academic center or those with increasing annualized esophagectomy volume was also associated with care at a single institution (Table 2).

### Survival Analysis

**Figure 3.**
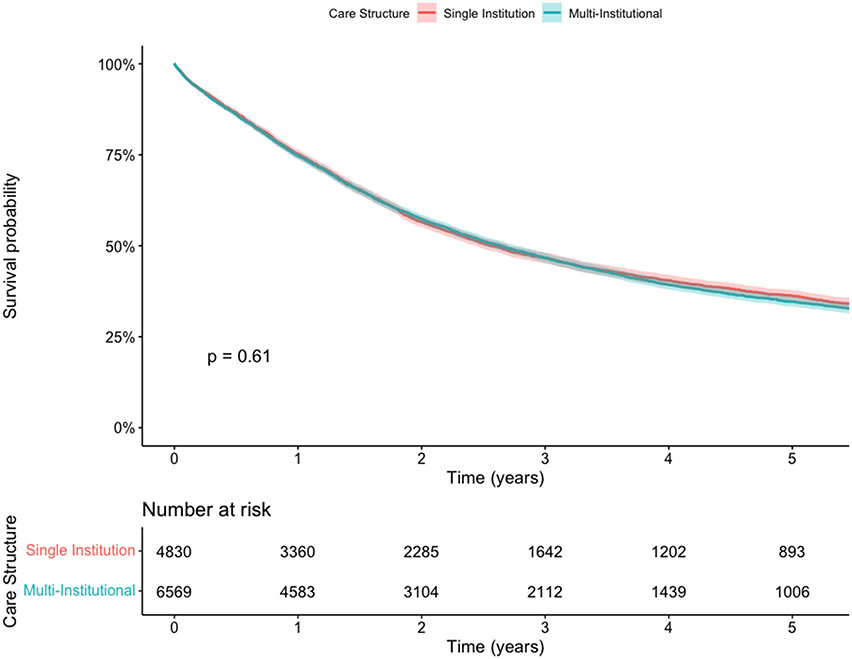
Kaplan Meier Survival Curves for patients with stage II/III esophageal cancer receiving neoadjuvant chemotherapy +/− radiation, stratified by care structure (single institution versus multi-institutional).

Median survival in the overall cohort was 2.62 (95% CI 2.51-2.7) years. When stratified by multi-institutional or single institution care, median survival was 2.64 (95% CI 2.5-2.76) and 2.56 (95% CI 2.44-2.72) years, respectively. Despite the differences in patient-, tumor-, and hospital-specific characteristics among groups noted above, patients who received multi-institutional care had comparable survival to those who received care at a single institution (adjusted HR 0.97, 95% CI 0.92-1.03; p=0.30) (Figure 3). When restricted to patients receiving neoadjuvant chemoradiation (N=10,369) and facility location for radiation was included in the definition of care structure, survival remained comparable (Supplemental Figure 1). Consistent with existing literature, increasing center annualized esophagectomy volume was associated with a survival benefit (adjusted HR 0.997, 95% CI 0.99-1.00; p=0.004).

### Secondary Outcomes

On adjusted multivariable analysis of secondary outcomes, multi-institutional care was not associated with higher rates of pathologic upstaging (adjusted OR 1.00, 95% CI 0.86-1.16; p=0.98) nor inferior guideline-concordant lymph node harvest (adjusted OR 0.97, 95% CI 0.89-1.06; p=0.47) compared to care at a single institution.

### Subgroup Analysis of Multi-Institutional Care

**Figure 4.**
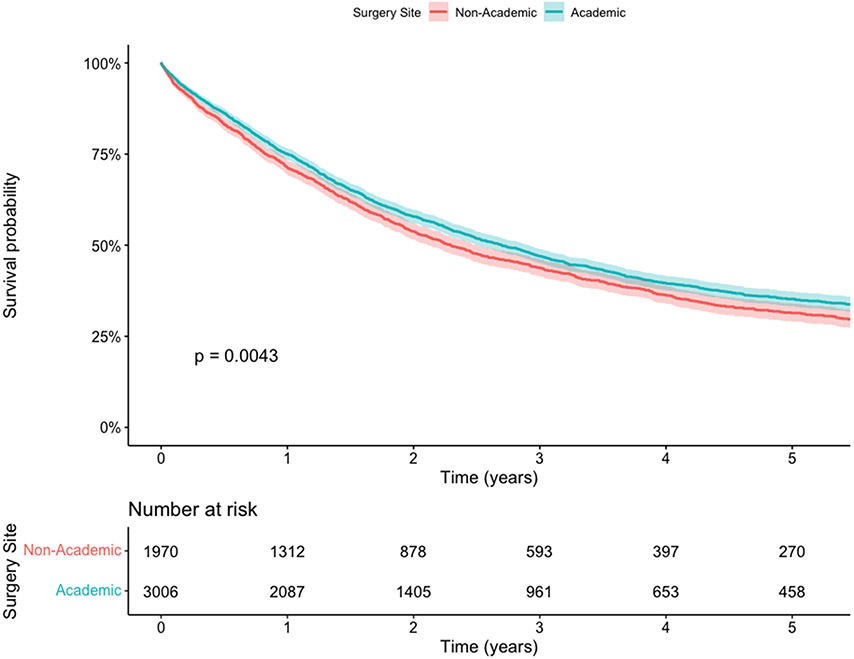

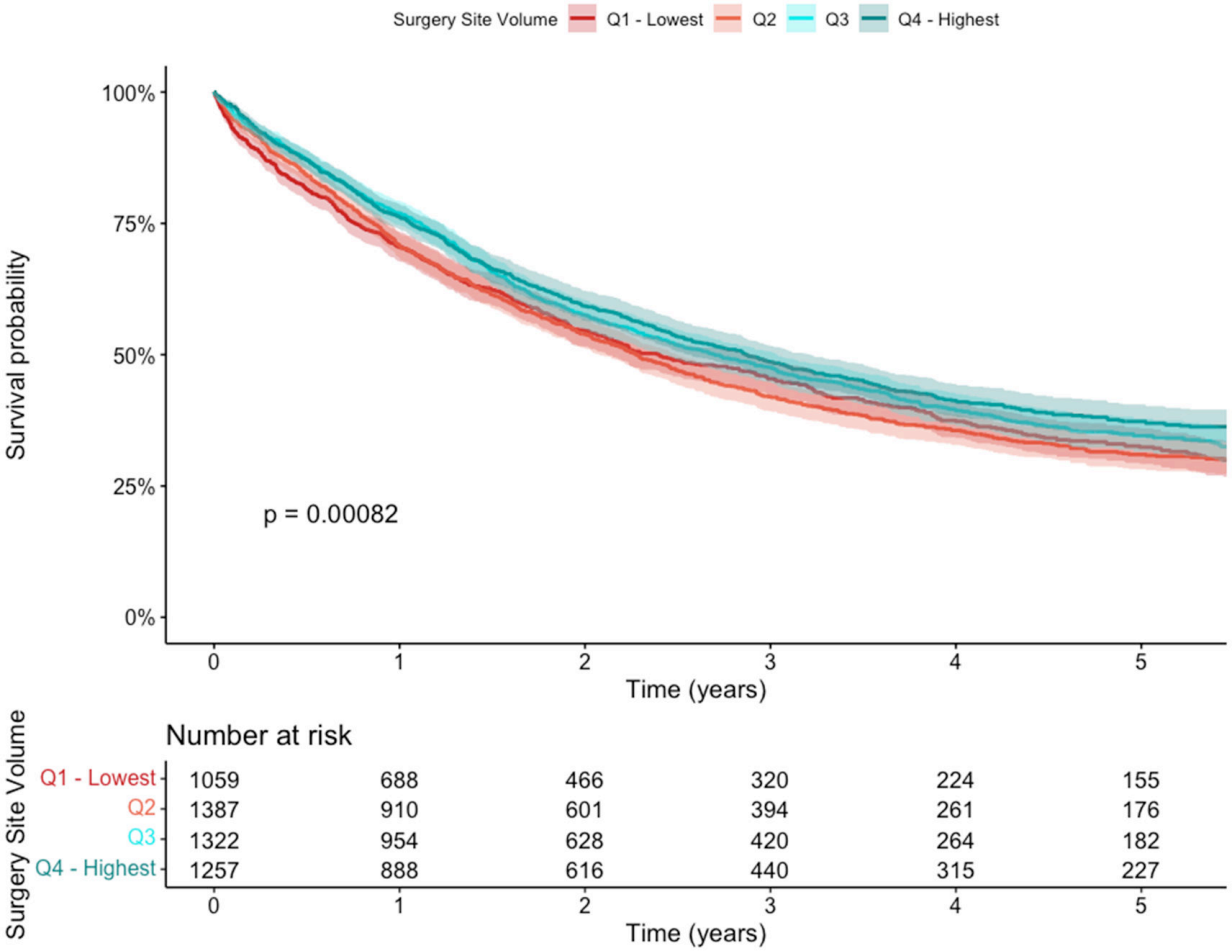
Kaplan Meier Survival Curves for patients with stage II/III esophageal cancer receiving neoadjuvant chemotherapy +/− radiation followed by surgery in a multi-institutional care pattern. A) Stratified by receipt of surgery at academic or non-academic site. B) Stratified by receipt of surgery at centers with increasing esophagectomy volume (Q1 ≤ 3 cases per year, Q2 3-8 cases per year, Q3 8-17 cases per year, Q4 >17 cases per year).

On subgroup analysis of patients receiving multi-institutional care (N=6,569), 5,025 (76.5%) received surgery at the reporting CoC-accredited facility with a median center esophagectomy volume of 8.2 per year (IQR 3.7-17.2). Of those patients, 3,006 (59.8%) were at academic centers. There was a survival benefit for patients who received the surgical component of their care at an academic center (Figure 4A) or a higher volume esophagectomy center (increasing by volume quartile) (Figure 4B). Patients receiving surgery at a high-volume academic center had the best overall survival, while there was no difference in survival for patients receiving surgery at low-volume academic or high-volume non-academic centers (Supplemental Figure 2). Within this subgroup of patients receiving multi-institutional care, median travel distance was farther for surgery (28.6 miles, IQR 10.6-68.9) than for chemotherapy (8.9 miles, IQR 4.2-19.2) (p<0.001).

## Discussion

This retrospective analysis of the NCDB is the first study, to our knowledge, to characterize multi-institutional care in patients with clinical stage II and III esophageal cancer. Multi-institutional care was highly prevalent among this cohort, and we describe many patient-, tumor-, and hospital-specific factors associated with this pattern of care. There was a marginal increase in time to surgery among patients receiving multi-institutional care, although this did not translate into inferior guideline-concordant nodal harvest nor higher rates of pathologic upstaging. Further, patients receiving multi-institutional care had comparable survival to those receiving care at a single institution, even when radiation was included in the definition. Notably, of patients with multi-institutional care, majority underwent surgery at academic or high-volume centers. Altogether, these findings suggest that patients receiving multidisciplinary therapy for esophageal cancer may be able to safely receive components of their care locally, while undergoing surgical resection at a high-volume center remains important.

Multi-institutional care among cancer patients has previously been described in other malignancies and highlighted as an area for improvement by the Institute of Medicine.(20) In the context of prior studies, the prevalence of multi-institutional care in our evaluation of esophageal cancer (57.6%) was equivalent to patients undergoing neoadjuvant therapy for pancreatic adenocarcinoma (57.6%), with decreasing prevalence in other malignancies as follows: gastric cancer (48.7%), ovarian cancer (36.8%), rectal cancer (35%), and hepatocellular carcinoma (27.4%).(14-17, 25) The differences in multi-institutional care among these disease sites is likely multifactorial, including factors such as disease prevalence and overall familiarity with treatment guidelines, need for multidisciplinary therapy, surgical expertise that requires travel to high-volume centers, and underlying disease biology.

Our study corroborates descriptions of multi-institutional care in other cancer populations. Findings from a retrospective analysis of locally advanced rectal cancer in the NCDB are consistent with ours, and suggest that multi-institutional care is more common among white patients, those with insurance, and farther travel distance for treatment.(14) Alternatively, patients receiving care at a single institution are more commonly black and uninsured. A study of hepatocellular carcinoma (HCC) within the Texas State Registry found similar patient-specific factors associated with receipt of multi-institutional care; however, in their study, multi-institutional care was more common among academic centers.(15) Consistent with our findings, they describe higher rates of multi-institutional care among patients residing in non-metropolitan areas; however, our study provides further insight on broader geographic regions associated with this pattern of care.(15) Overall, our findings in conjunction with the existing literature suggest a dichotomy in the patients who receive multi-institutional care. For example, patients with financial resources to travel to high-volume cancer centers are likely distinct from those who receive multi-institutional care at lower-volume facilities out of necessity due to low socioeconomic status, inability to take time off work, or their rural residence. Further work is needed to better define these populations receiving multi-institutional care and its potential role as a health disparity.

While patient- and hospital-specific factors associated with multi-institutional care are largely consistent, the impact of multi-institutional care on receipt of timely therapy and survival is highly variable. We describe a longer interval between neoadjuvant therapy and surgery among patients with multi-institutional care; however, this three-day difference is not clinically significant and there was no association with survival. Outside of esophageal cancer, multi-institutional care has been associated with inferior survival in both locally advanced rectal cancer, HCC, and our own group’s analysis of gastric cancer.(14, 15, 25) However, this relationship appears more complex among malignancies with classically aggressive tumor biology. In an analysis of pancreatic adenocarcinoma, there was an increase in time to neoadjuvant therapy among patients receiving multi-institutional care, although there was no difference in overall survival.(16) Similarly, this care structure has been associated with delays in adjuvant therapy after debulking for ovarian cancer, but was not detrimental to long-term mortality.(17) Altogether, these findings suggest that the association of multi-institutional care with survival is not universal. Perhaps, care at a single institution has an impact on the receipt of timely therapy but is not able to overcome aggressive disease biology.

Importantly, our study supports the mounting literature regarding the volume-outcomes relationship for esophagectomy and survival in esophageal cancer. We found that patients receiving multi-institutional care travel farther distances for surgery compared to chemotherapy. Among those patients, the median center esophagectomy volume was consistent with that observed in the single institution group (8.2 per year) and there was a survival benefit with receiving the surgical component of their care at an academic or higher volume center. Overall, this supports continued referral of patients with resectable disease to high-volume esophagectomy centers and suggests that receiving chemotherapy, with or without radiation, locally does not compromise outcomes.

Limitations of this study include those inherent to retrospective observational studies. For instance, selection bias exists for which we cannot fully adjust for; we cannot account for provider or patient preferences in neoadjuvant therapy nor where they receive their care. Additionally, we are limited by the lack of granularity in a large national dataset such as the NCDB. Importantly, the NCDB does not provide information on how patients are staged, chemotherapy agents utilized, or cycles completed. Further, the NCDB does not provide hospital-specific information on the secondary institution in which a patient received multi-institutional care. Additionally, our study only captures those who were able to receive neoadjuvant therapy and undergo surgical resection. There are likely some patients who become lost to follow-up in the setting of multi-institutional care and may have poor outcomes. With these limitations in mind, our group is conducting an institutional review of patients with esophageal and gastric cancer to further define the population that receives multi-institutional care and their outcomes. In addition, we hope to identify potential mechanisms to improve care coordination in the multidisciplinary setting and among satellite institutions.

## Conclusions

Altogether, this NCDB analysis represents the first step in characterizing multi-institutional care among patients with clinical stage II and III esophageal cancer and contributes to the mounting literature on this pattern within broader cancer care. During the study period, a significant number of patients received neoadjuvant chemotherapy with or without radiation at a different facility than where they ultimately underwent esophagectomy; however, in this cohort of patients that completed multidisciplinary therapy, this pattern of care did not have an adverse relationship with survival. In this era of increasing centralization of complex cancer care, thoracic surgeons should continue to advocate for referral to high-volume surgical centers for esophagectomy while patients may consider receiving other components of their multidisciplinary care closer to home.

## Supplementary Material

Supplementary

## Figures and Tables

**Table 1.** Baseline Patients Demographics for patients with clinical stage II/III esophageal cancer receiving neoadjuvant therapy prior to resection.

Variable	Single Institution Care N = 4830 (%)	Multi-Institutional Care N = 6569 (%)	p-value
Age (years, median [IQR])	62 [56, 68]	63 [56, 69.0]	0.028
Sex			0.100
Male	4089 (84.7)	5485 (83.5)	
Female	741 (15.3)	1084 (16.5)	
Race			<0.001
White	4439 (92.6)	6174 (94.8)	
Black	257 (5.4)	219 (3.4)	
Other	96 (2.0)	120 (1.8)	
Year of Diagnosis (median [IQR])	2011 [2008, 2013]	2012 [2008, 2014]	<0.001
CD Comorbidity Score			0.475
0	3535 (73.2)	4740 (72.2)	
1	1021 (21.1)	1443 (22.0)	
2+	274 (5.7)	386 (5.9)	
Insurance Status			<0.001
Private	2340 (50.0)	3234 (49.8)	
Government	2191 (46.8)	3149 (48.5)	
None	148 (3.2)	112 (1.7)	
Medicaid Expansion State Status *Only patients diagnosed 2010+			<0.001
No Expansion	961 (32.7)	1736 (39.1)	
Early Expansion	402 (13.7)	591 (13.3)	
Late Expansion (2014+)	1578 (53.7)	2114 (47.6)	
Patient Residence			<0.001
Metro	3847 (82.2)	5007 (78.3)	
Urban	742 (15.9)	1223 (19.1)	
Rural	92 (2.0)	168 (2.6)	
Distance to Treating Facility (miles, median [IQR])	15.8 [6.9, 39.0]	20.9 [7.7, 56.6]	<0.001
Education Quartile (% completing high school)			0.001
Q1 (>21%)	594 (12.4)	754 (11.5)	
Q2 (13-20.9%)	1194 (24.8)	1702 (26.0)	
Q3 (7-12.9%)	1681 (35.0)	2462 (37.6)	
Q4 (<7%)	1340 (27.9)	1630 (24.9)	
Income Quartile			0.001
Q1 (<$38,000)	697 (14.5)	937 (14.3)	
Q2 ($38,000-47,999)	1105 (23.0)	1641 (25.1)	
Q3 ($48,000-62,999)	1326 (27.6)	1898 (29.0)	
Q4 (>$63,000)	1680 (34.9)	2069 (31.6)	
Facility Region			<0.001
New England ^(CT, MA, ME, NH, RI, VT)^	351 (7.4)	589 (9.1)	
Middle Atlantic ^(NJ, NY, PA)^	971 (20.5)	876 (13.5)	
South Atlantic ^(DC, DE, FL, GA, MD, NC, SC, VA, WV)^	932 (19.6)	1455 (22.4)	
East North Central ^(IL, IN, MI, OH, WI)^	926 (19.5)	1270 (19.6)	
East South Central ^(AL, KY, MS, TN)^	211 (4.4)	413 (6.4)	
West North Central ^(IA, KS, MN, MO, ND, NE, SD)^	621 (13.1)	758 (11.7)	
West South Central ^(AR, LA, OK, TX)^	218 (4.6)	363 (5.6)	
Mountain ^(AZ, CO, ID, MT, NM, NV, UT, WY)^	249 (5.2)	212 (3.3)	
Pacific ^(AK, CA, HI, OR, WA)^	267 (5.6)	552 (8.5)	
Academic Center	3078 (64.9)	3262 (50.3)	<0.001
Facility Esophagectomy Volume (per year, median [IQR])	8.2 [3.1, 18.5]	5.2 [1.4, 14.1]	<0.001
Radiation Therapy			0.053
None	262 (5.4)	347 (5.3)	
Neoadjuvant	4363 (90.3)	6006 (91.4)	
Adjuvant	174 (3.6)	179 (2.7)	
Perioperative	31 (0.6)	37 (0.6)	
Clinical Stage			0.007
II	2176 (45.1)	3127 (47.6)	
III	2654 (54.9)	3442 (52.4)	
Time to Surgery (days, median [IQR])	92 [79, 110]	95 [83, 114]	<0.001
Pathologic Stage			0.069
I	1379 (39.8)	2046 (42.7)	
II	1218 (35.1)	1580 (33.0)	
III	707 (20.4)	937 (14.3)	
IVa	102 (2.9)	156 (2.4)	
IVb	61 (1.8)	75 (1.6)	
Pathologic Upstaging	367 (7.6)	528 (8.0)	0.408
Histology			0.005
Adenocarcinoma	3903 (80.8)	5443 (82.9)	
Squamous Cell Carcinoma	927 (19.2)	1126 (17.1)	
Grade			0.004
Low	224 (5.4)	270 (4.9)	
Moderate	1891 (45.2)	2583 (46.6)	
High	2070 (49.5)	2693 (48.6)	
Guideline-Concordant Lymph Node Harvest (15+)	1962 (40.6)	2475 (37.7)	0.002
Hospital Length of Stay (days, median [IQR])	10 [8, 14]	9 [7, 14]	<0.001
30-Day Readmission	296 (6.1)	315 (4.8)	0.002

**Table 2.** Multivariable logistic regression of factors associated with receipt of multi-institutional care in patients with clinical stage II/III esophageal cancer receiving neoadjuvant therapy and surgical resection.

Variable	Odds Ratio	95% Confidence Interval	p-value
Age (per year)	1.00	0.99-1.01	0.98
Female Sex (reference: male)	1.14	1.02-1.28	0.02
Race (reference: White)			
Black	0.63	0.51-0.78	<0.001
Other	1.11	0.84-1.55	0.38
Year of diagnosis (per year)	1.04	1.03-1.06	<0.001
CDCC Score (reference: 0)			
1	1.04	0.95-1.15	0.39
≥2	1.02	0.86-1.21	0.84
Insurance (reference: private)			
Government	0.98	0.89-1.08	0.73
None	0.49	0.37-0.64	<0.001
Patient Residence (reference: metro)			
Urban	1.22	1.09-1.37	<0.001
Rural	1.33	1.00-1.77	0.05
Academic center	0.81	0.73-0.90	<0.001
Education quartile (reference: Q1 >21%)			
Q2 (13-20.9%)	1.13	0.97-1.31	0.12
Q3 (7-12.9%)	1.29	1.10-1.52	0.002
Q4 (<7%)	1.19	0.99-1.43	0.07
Income quartile (reference: Q1 <$38,000)			
Q2 ($38,000-47,999)	1.03	0.90-1.19	0.64
Q3 ($48,000-62,999)	1.03	0.89-1.19	0.68
Q4 (>$63,000)	0.93	0.78-1.10	0.38
Facility location (reference: New England ^(CT,MA,ME,NH,RI,VT)^)			
Middle Atlantic ^(NJ, NY, PA)^	0.63	0.53-0.75	<0.001
South Atlantic ^(DC, DE, FL, GA, MD, NC, SC, VA, WV)^	1.01	0.85-1.20	0.89
East North Central ^(IL, IN, MI, OH, WI)^	0.83	0.70-0.98	0.03
East South Central ^(AL, KY, MS, TN)^	1.17	0.93-1.47	0.19
West North Central ^(IA, KS, MN, MO, ND, NE, SD)^	0.74	0.61-0.89	0.002
West South Central ^(AR, LA, OK, TX)^	1.51	1.18-1.94	0.001
Mountain ^(AZ, CO, ID, MT, NM, NV, UT, WY)^	0.43	0.34-0.55	<0.001
Pacific ^(AK, CA, HI, OR, WA)^	1.11	0.90-1.38	0.33
Clinical Stage (reference: stage II)	0.96	0.88-1.04	0.28
Squamous Histology (reference: adenocarcinoma)	0.88	0.79-0.99	0.03
Neoadjuvant RT (reference: no neoadjuvant RT)	1.12	0.97-1.28	0.12
Distance Traveled to Facility (per mile)	1.001	1.000-1.001	<0.001
Center Esophagectomy Volume (increasing per year)	0.73	0.69-0.77	<0.001
Medicaid Expansion State Status (reference: no expansion) *Only patients diagnosed after 2010			
Early Expansion	0.75	0.61-0.93	0.008
Late Expansion (2014+)	0.80	0.69-0.93	0.003

## Abbreviations:

CoC: Commission on Cancer
NCDB: National Cancer Database
NCCN: National Comprehensive Cancer Network
HCC: Hepatocellular Carcinoma
