# Supplementary material for: Multi-Institutional Care in Clinical Stage II and III Esophageal Cancer
Source: Ann Thorac Surg. Author manuscript; Available in PMC 2024 Feb 1. (PMC9851933; doi:10.1016/j.athoracsur.2022.06.049)

**Supplemental Figure 1.** Kaplan Meier Survival Curves for patients with stage II/III esophageal cancer receiving neoadjuvant chemoradiation followed by surgery in a multi-institutional care pattern, where facility of radiation therapy is included in the definition of care structure.


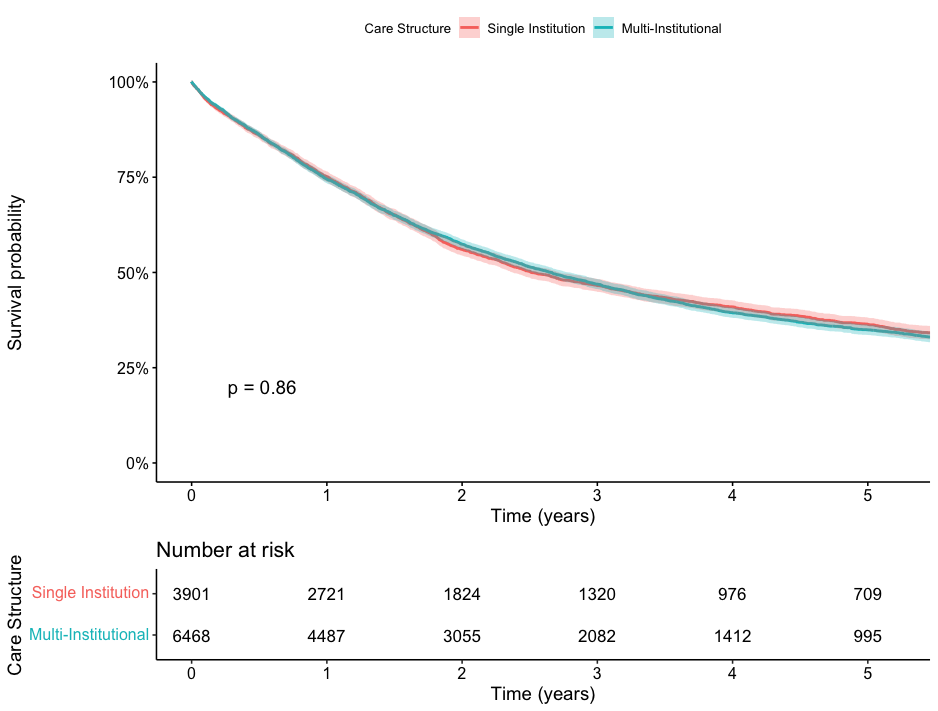


**Supplemental Figure 2.** Kaplan Meier Survival Curves for patients with stage II/III esophageal cancer receiving neoadjuvant chemotherapy +/- radiation followed by surgery in a multi-institutional care pattern. A) Stratified by surgical site academic and volume status, with high-volume defined as greater than the median cases in the multi-institutional cohort (roughly 8 cases per year). B) Comparison of only low-volume academic and high-volume non-academic curves.

A)


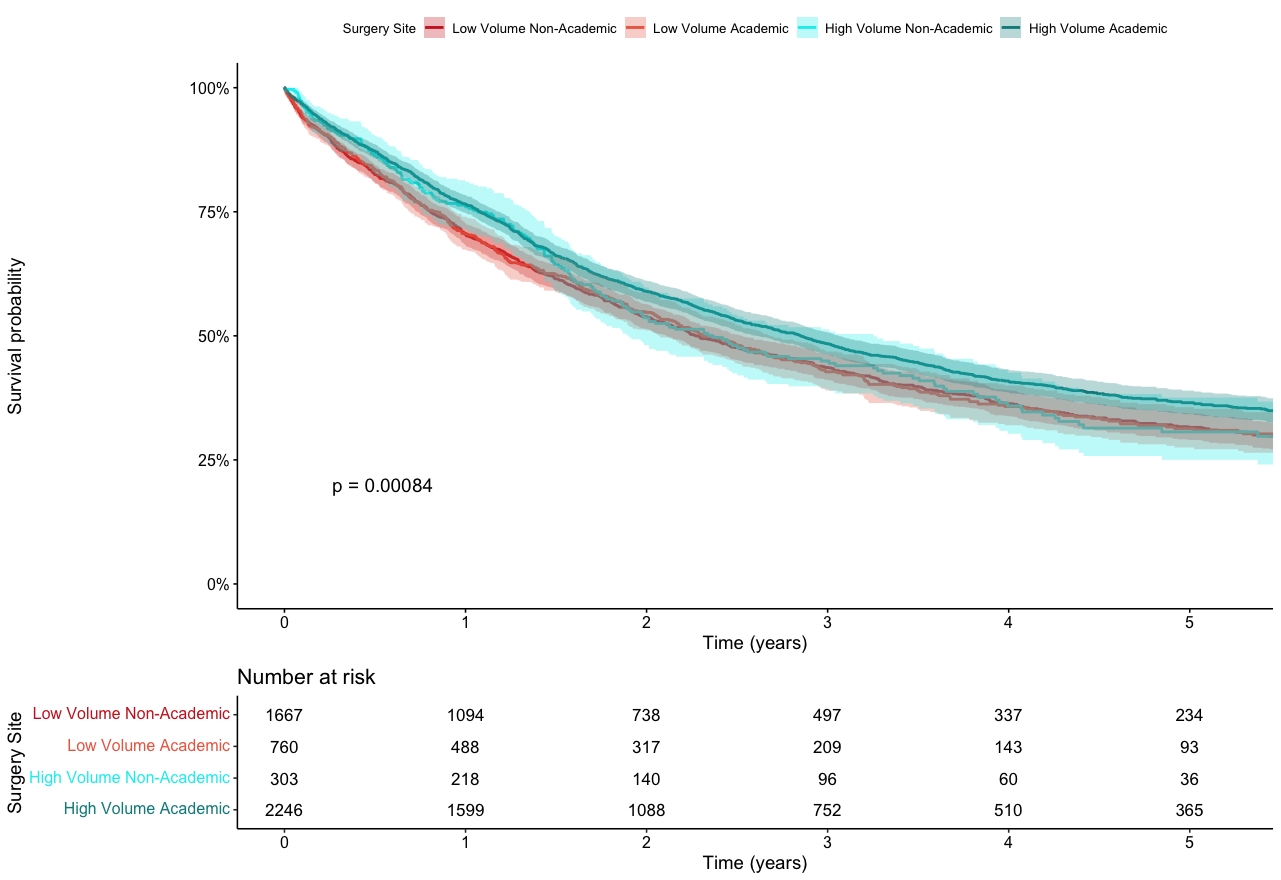


B)
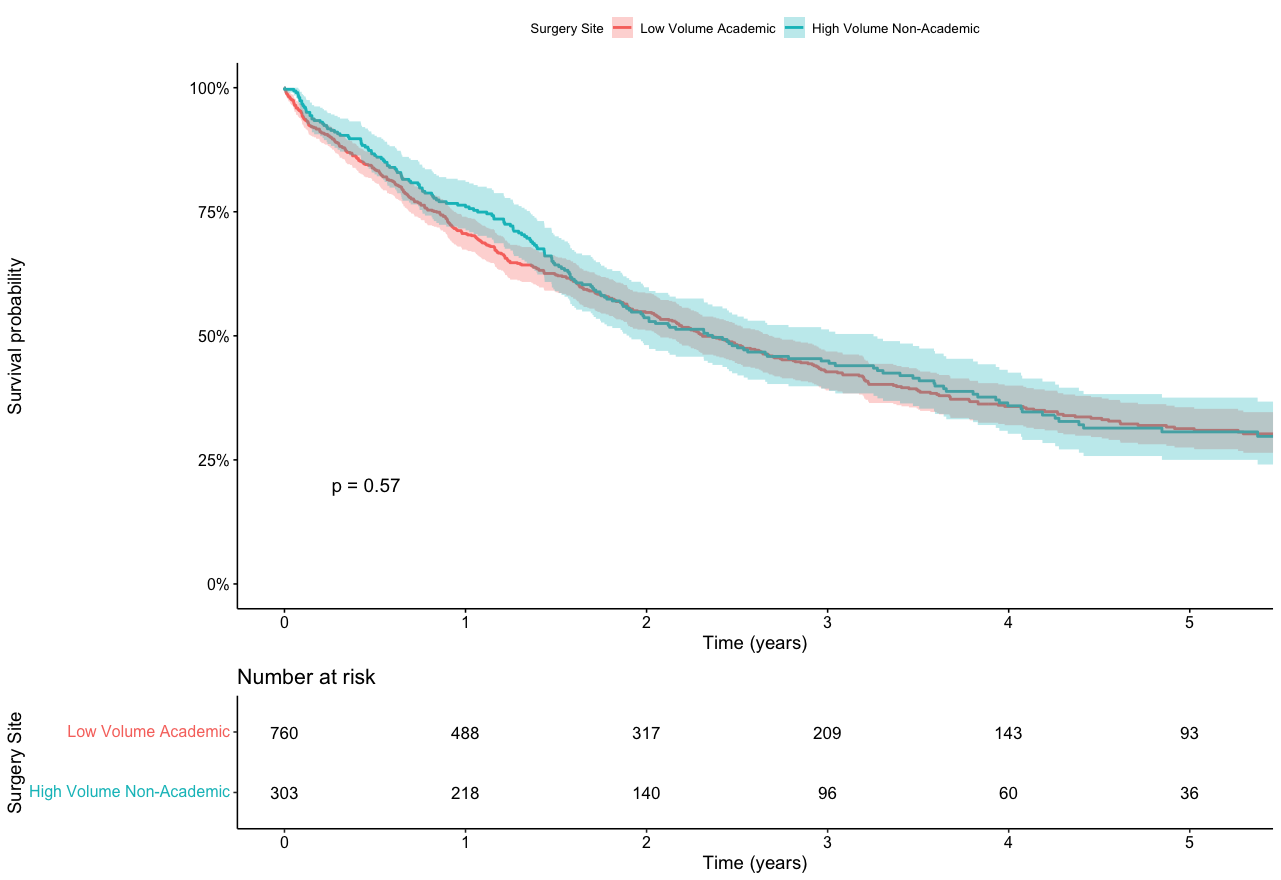

Supplement: Supplementary [file NIHMS1829969-supplement-Supplementary.docx]
